# rs1051838 Promotes Intracellular Survival of *Mycobacterium tuberculosis* H37Ra by Regulating *DUSP14* Expression

**DOI:** 10.3390/microorganisms14071588

**Published:** 2026-07-21

**Authors:** Jiao Feng, Yan Meng, Jinyu Liang, Yuming Zhang, Runru Wang, Yanran Li, Xiaogang Cui, Zhiqiang Yang, Li Xing, Changxin Wu

**Affiliations:** Biomedicine and Health Laboratory in Shanxi Province, Institute of Biomedical Sciences, Shanxi University, Taiyuan 030006, China; fengjiao-1219@163.com (J.F.); my990925@163.com (Y.M.); 19834031730@163.com (J.L.); ymzhang202512@163.com (Y.Z.); wangrnuru@163.com (R.W.); lyr3121007@163.com (Y.L.); xgcui@sxu.edu.cn (X.C.); yzq2021@sxu.edu.cn (Z.Y.)

**Keywords:** tuberculosis, DUSP14, rs1051838, methylation, JNK

## Abstract

Tuberculosis (TB), caused by *Mycobacterium tuberculosis* (*Mtb*), remains a major global health burden. Host genetic factors play a critical role in TB susceptibility, but the underlying mechanisms are not fully understood. Genome-wide association studies have identified *DUSP14* as a TB susceptibility gene, and the single nucleotide polymorphism (SNP) rs1051838 in *DUSP14* seems to be associated with TB risk. Using in vitro models including THP-1-derived macrophages, A549 cells, and HEK293T cells, we investigated how rs1051838 regulates *DUSP14* expression and modulates macrophage responses to *Mtb* infection. We found that the G allele of rs1051838 exhibits higher transcriptional activity than the A allele in reporter assays. The G allele creates a CpG dinucleotide that is subject to methylation-mediated repression. *Mtb* infection reduced CpG methylation across this region to upregulate DUSP14, leading to suppressed JNK phosphorylation via its phosphatase activity, reduced pro-inflammatory cytokine production, and promoted intracellular bacterial survival. Knockdown of *DUSP14* decreases bacterial burden and increases cytokine secretion. These findings reveal a functional link between genetic variation and immune response in TB and point to DUSP14 as a potential target for host directed therapy, although further validation in primary cells or using virulent strains and in vivo models is needed.

## 1. Introduction

Tuberculosis (TB), caused by *Mycobacterium tuberculosis* (*Mtb*), remains a major global health threat. The World Health Organization estimated approximately 10.7 million new TB cases in 2024 [1]. Upon exposure to *Mtb*, only about one-quarter of individuals become infected, and among them, approximately 10% develop active pulmonary TB [2,3]. This phenomenon suggests that host genetic factors play a critical role in determining the outcome of *Mtb* infection, yet the underlying mechanisms remain incompletely understood.

Genome-wide association studies (GWAS) have identified numerous susceptibility loci associated with TB risk, including the gene encoding dual-specificity phosphatase 14 (DUSP14) [4,5,6,7,8,9]. DUSP14, also known as MAPK phosphatase 6 (MKP6), is an atypical dual-specificity phosphatase that negatively regulates MAPK signaling by directly dephosphorylating ERK and JNK [10,11]. In addition, DUSP14 can inactivate TAB1 by dephosphorylating its Ser438 residue, thereby inhibiting TAK1 activation and downstream JNK/IKK signaling [12]. Thus, DUSP14 functions as an important negative regulator to prevent excessive immune activation. However, how DUSP14 modulates macrophage responses during *Mtb* infection has not been systematically investigated.

A number of single nucleotide polymorphisms (SNPs) have been reported to be associated with TB, most of which are located in non-coding regions of the genome. Emerging evidence suggests that such non-coding SNPs may affect gene expression by altering transcription factor binding, DNA methylation, or miRNA interactions [13,14,15]. One non-coding SNP in DUSP14, rs1051838, is located in the 5′-untranslated region (5′-UTR) of exon 2. The 5′-UTR is known to regulate gene expression through various mechanisms, including modulation of transcription factor binding and DNA methylation within CpG islands [16]. Previous studies have identified *DUSP14* as a response eQTL in primary dendritic cells and reported associations between rs1051838 genotypes and *DUSP14* mRNA levels in whole blood [8,9]. However, the functional mechanisms through which rs1051838 regulates *DUSP14* transcription remained unknown.

In this study, we investigated the regulatory effects of rs1051838 on the expression of *DUSP14* and the modulation of macrophage response to *Mtb* infection. The results reveal that rs1051838 may function as a CpG-SNP to regulate *DUSP14* transcription by changing methylation status, and that the upregulation of DUSP14 promotes intracellular *Mtb* survival by suppressing JNK phosphorylation and pro-inflammatory cytokine production.

## 2. Materials and Methods

### 2.1. Cell Culture

HEK293T, A549 and THP-1 cell lines were obtained from the American Type Culture Collection (ATCC). HEK293T and A549 cells were cultured in DMEM medium (Boster, Wuhan, China) supplemented with 10% fetal bovine serum (FBS; Gibco, Thermo Fisher Scientific, Waltham, MA, USA) and 1% penicillin–streptomycin solution (Solarbio, Beijing, China). THP-1 cells were maintained in RPMI-1640 medium (Gibco, Thermo Fisher Scientific, Waltham, MA, USA) supplemented with 10% FBS and 1% penicillin–streptomycin. All cell lines were incubated at 37 °C in a humidified atmosphere with 5% CO_2_. Cells were used between passages 5 and 15 for THP-1 and A549, and between passages 3 and 10 for HEK293T. For differentiation into adherent macrophage-like cells, THP-1 cells were seeded at a density of 1 × 10^6^ cells/well in 6 well plates and incubated with 100 ng/mL phorbol 12 myristate 13 acetate (PMA; Sigma Aldrich, St. Louis, MO, USA) for 48 h prior to infection or transfection experiments.

### 2.2. Genotyping of rs1051838 in THP-1 and A549 Cells and Bioinformatics Analysis

The genotype of rs1051838 in THP-1 and A549 cells were determined by Sanger sequencing. The primer sequences are listed in Supplementary Table S1. To predict whether rs1051838 affects transcription factor binding, a 25 bp sequence encompassing rs1051838 (12 bp upstream and 12 bp downstream of the SNP) was submitted to the JASPAR database (https://jaspar.elixir.no/, JASPAR 2024 release) using the JASPAR Scan tool.

### 2.3. DNA Methylation Analysis

Genomic DNA was extracted from THP-1 and A549 cells using a DNA extraction kit (Tiangen, Beijing, China). Bisulfite conversion was performed using the EpiTect Bisulfite Kit (Qiagen, Hilden, Germany). The converted DNA was amplified with primers targeting rs1051838 (M-rs1051838-F/M-rs1051838-R; primer sequences are listed in Supplementary Table S1). The PCR products were inserted into the pMD19-T vector (TaKaRa, Kyoto, Japan). After transformation into *Escherichia coli* DH5α cells, 20–30 colonies per sample were randomly picked, and 10 positive clones per sample were sequenced. Methylation status of CpG sites was analyzed using the online Quantification Tool (https://quma.cdb.riken.jp/, accessed on 26 April 2024) for Methylation Analysis (QUMA).

### 2.4. Effect of DNA Methylation on DUSP14 Expression

To determine whether DNA methylation regulates DUSP14 expression, THP-1 and A549 cells were treated with 5 µM Decitabine (a DNA methyltransferase inhibitor; MedChemExpress, Monmouth Junction, NJ, USA) for 72 h. Cells cultured in normal medium served as controls. After treatment, total RNA was extracted and subjected to RT-qPCR to measure DUSP14 transcript levels.

### 2.5. Dual-Luciferase Reporter Gene Assay

To construct the reporter plasmids, the 349 bp fragment encompassing rs1051838 was amplified from genomic DNA of THP-1 or A549 cells using specific primers (primer sequences are listed in Supplementary Table S1). The purified PCR products were digested with SacI and HindIII, and then inserted into pGL3-basic vector to generate recombinant plasmids pGL3-rs1051838-G and pGL3-rs1051838-A. Plasmids were verified by Sanger sequencing. To assess the effect of methylation, the pGL3-rs1051838-G plasmid was subjected to in vitro methylation using CpG methyltransferase M.SssI (New England Biolabs, Hitchin, UK).

For the dual-luciferase assay, HEK293T cells were co-transfected with the indicated reporter plasmid and pRL-TK (as an internal control) using Lipofectamine 3000 (Thermo Fisher Scientific, Waltham, MA, USA). At 48 h post-transfection, cells were lysed, and luciferase activities were measured using a dual-luciferase reporter assay kit (TransGen Biotech, Beijing, China) according to the manufacturer’s protocol. All experiments were performed in triplicate.

### 2.6. H37Ra Infection and Optimization of Infection Conditions

*Mtb* H37Ra (provided by Professor Jian Gao, Fudan University, Shanghai, China) was used for all infection experiments. Initially, THP-1-derived macrophages were infected with H37Ra at multiplicities of infection (MOI) of 1, 5, or 10 for 6 h to determine the optimal infection conditions. According to these results, subsequent time-course analyses were performed at an MOI of 10 for 6, 12, or 24 h. After infection, cells were harvested for measure DUSP14 mRNA and protein levels by real-time quantitative PCR (RT-qPCR) and Western blotting, respectively.

### 2.7. Real-Time Quantitative PCR

Total RNA was extracted using RNAiso Plus (TaKaRa, Kyoto, Japan) and its concentration was determined using a Nanodrop 2000 spectrophotometer (Thermo, Waltham, MA, USA). Complementary DNAs (cDNAs) were synthesized using the PrimeScriptTM RT Master Mix (TaKaRa, Kyoto, Japan) following the procedures outlined in the instructions. RT-qPCR was performed with 2 × Universal SYBR Green qPCR Master Mix (Servicebio, Wuhan, China) on a Light Cycler 480 system (Roche, Basel, Switzerland). Each sample was run in triplicate. The primer sequences are shown in Supplementary Table S1. The qPCR program consisted of an initial denaturation at 95 °C for 30 s, followed by 40 cycles of 95 °C for 15 s and 55 °C for 30 s. Relative DUSP14 mRNA expression was calculated using the 2^−ΔΔCt^ method. All primers were purchased from Sangon Biotechnology Co. (Shanghai, China).

### 2.8. Western Blotting

Cell lysates were prepared using RIPA lysis buffer (Solarbio, Beijing, China) supplemented with 1% phenylmethanesulfonyl fluoride (PMSF; Solarbio, Beijing, China) and 1% phosphatase inhibitor cocktail I (GLPBIO, Montclair, NJ, USA) on ice. Protein concentrations were determined using a BCA protein assay kit (Solarbio, Beijing, China). Equal amounts of protein (20 µg) were separated by SDS-PAGE and transferred onto polyvinylidene fluoride (PVDF) membranes (0.45 µm). The membranes were blocked using 2.5% fat-free milk in Tris-buffered saline containing 0.1% Tween-20 (TBST) for 2 h at 4 °C, and then incubated overnight at 4 °C with primary antibodies. After washing, the membranes were incubated with horseradish peroxidase (HRP)-conjugated goat anti-rabbit IgG secondary antibody (AC026, ABclonal, Wuhan, China; 1:5000 dilution) for 1 h at 37 °C. Protein bands were visualized by chemiluminescence using SuperKing™ Hypersensitive luminescent ELC solution (Abbkine, Beijing, China) and quantified using ImageJ software (version 1.48v). The primary antibodies used were as follows: anti-DUSP14 (A10287, ABclonal, Wuhan, China; 1:1000 dilution), anti-JNK (51153-1-AP, Proteintech, Wuhan, China; 1:1000 dilution), and anti-P-JNK (R381100, Zenbio, Chengdu, China; 1:1000 dilution).

### 2.9. Transient Transfection and Stable Cell Line Construction

For transient transfection, siRNAs were delivered into cells using Lipofectamine 3000 (Thermo Fisher Scientific, Waltham, MA, USA) according to the manufacturer’s protocol. The siRNA sequences are listed in Supplementary Table S1 and were synthesized by Sangon Biotechnology Co. (Shanghai, China). For stable overexpression, THP-1 cells were transduced with lentivirus carrying the DUSP14 expression cassette. After 24 h of infection, the cells were cultured in fresh medium containing puromycin (1 μg/mL) for 2 weeks to select stably transfected clones. The lentivirus was packaged and provided by HyCyte (Suzhou, China).

### 2.10. PTP Inhibitor IV Treatment

To inhibit DUSP14 phosphatase activity, THP-1-derived macrophages were pretreated with PTP Inhibitor IV (MedChemExpress, NJ, USA) at a concentration of 50 µM for 30 min, followed by H37Ra infection (MOI = 10). Cells treated with an equal volume of DMSO served as vehicle controls. After treatment, cells were harvested at indicated time points (0, 15, 30, 60, and 120 min) for Western blot analysis of JNK phosphorylation. All experiments were performed in triplicate.

### 2.11. Colony-Forming Unit (CFU) Assay

THP-1-derived macrophages were infected with H37Ra at a MOI of 10 for 24 h. After infection, cells were incubated with gentamicin (100 µg/mL) for 2 h and then washed three times with PBS and lysed in PBS containing 0.1% Triton-X 100 (Solarbio, Beijing, China). Serial dilutions (10-fold and 100-fold) of the lysates were prepared, and 60 μL of each dilution was plated onto Middlebrook 7H10 agar plates (BD, Franklin Lakes, NJ, USA) supplemented with 10% OADC enrichment (BD, NJ, USA). The plates were incubated at 37 °C for 4 weeks, and CFUs were counted.

### 2.12. Measurement of Cytokine Secretion by Enzyme-Linked Immunosorbent Assay (ELISA)

The levels of IL-1β, IL-6 and TNF-α in cell culture supernatants were measured using ELISA kits EK0392, EK0410, and EK0525, respectively (BOSTER, Wuhan, China). After *Mtb* infection, supernatants were collected and centrifuged at 4000 rpm for 10 min to remove particles. The cleared supernatants were diluted 5-fold and 100 μL of each sample was added to the ELISA plate. Subsequently, 100 μL of horseradish peroxidase (HRP)-conjugated reagent was added to each well, and the plate was sealed with an adhesive strip and incubated at 37 °C for 60 min. After five washes with wash buffer (1 min each), 50 μL of chromogen solution A and 50 μL of chromogen solution B were added to each well, mixed gently, and incubated at 37 °C for 15 min in the dark. The reaction was terminated by adding 50 μL of stop solution, and the absorbance was measured at 450 nm using a microplate reader (H1M, BioTek, Winooski, VT, USA).

### 2.13. Statistical Analysis

All experiments were performed with at least three independent biological replicates. Data are presented as mean ± standard deviation (SD). Statistical analyses were carried out using GraphPad Prism 8.00. Normality and variance homogeneity were assessed prior to parametric testing. Comparisons between two groups were analyzed using Student’s *t*-test, while comparisons among multiple groups were performed using one-way analysis of variance (ANOVA) followed by Tukey’s post hoc test. A *p* value < 0.05 was considered statistically significant (ns, *p* > 0.05; *, *p* < 0.05; **, *p* < 0.01; ***, *p* < 0.001; and ****, *p* < 0.0001).

## 3. Results

### 3.1. rs1051838 Regulates DUSP14 Expression in an Allele-Specific Manner

**Figure 1:**
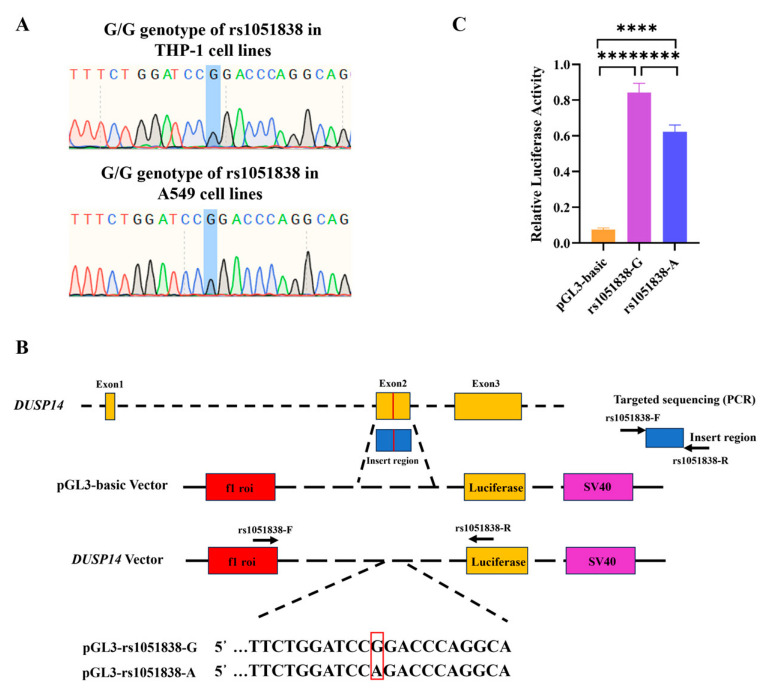
rs1051838 regulates DUSP14 expression in an allele-specific manner. (**A**) Genotyping of rs1051838 in THP-1 and A549 cells by Sanger sequencing. Both cell lines carry the homozygous G/G genotype. Results are representative of three independent experiments. (**B**) Schematic representation of the reporter constructs. The pGL3-basic vectors carry either the G or A allele of rs1051838 within a 349-bp genomic fragment. The red box indicates the position of rs1051838. (**C**) Dual-luciferase reporter assay of rs1051838-G or rs1051838-A allele in HEK293T cells. Both alleles enhanced luciferase activity compared with the empty vector, with the G allele showing significantly higher activity than the A allele. Data are presented as mean ± SD from three independent experiments (*n* = 3). ****, *p* < 0.0001.

Bioinformatics analysis predicted that rs1051838 is located within a potential transcription factor binding region, and the G and A alleles may differentially affect transcription factor binding affinity (Supplementary Tables S2 and S3). To determine whether this locus is functionally relevant, we first examined its genotype in THP-1 and A549 cell lines. Genotyping confirmed that both cell lines carry the homozygous G/G genotype at rs1051838 (Figure 1A).

We then performed dual-luciferase reporter assays to compare the transcriptional activities of the two alleles. Reporter vectors carrying either the G or A allele in a 349-bp fragment flanking rs1051838 were transfected into HEK293T cells (Figure 1B). Compared with the empty vector control, both alleles significantly enhanced luciferase activity. Importantly, the rs1051838-G allele drove significantly higher luciferase activity than the A allele (Figure 1C), indicating that the G allele possesses a stronger transcriptional activation capacity.

### 3.2. DNA Methylation Around rs1051838 Regulates DUSP14 Expression

**Figure 2:**
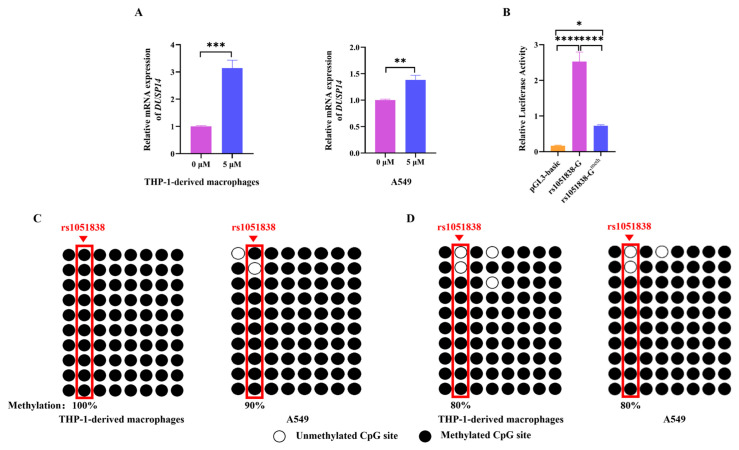
DNA methylation around rs1051838 regulates DUSP14 expression. (**A**) *DUSP14* mRNA levels in THP-1-derived macrophages and A549 cells treated with Decitabine compared with untreated controls. Data are presented as mean ± SD from three independent experiments (*n* = 3). (**B**) Dual-luciferase reporter assay of HEK293T cells transfected with unmethylated or in vitro-methylated pGL3-rs1051838-G. Data are presented as mean ± SD from three independent experiments (*n* = 3). (**C**) Methylation status of CpG sites in the region flanking rs1051838 in THP-1-derived macrophages and A549 cells under basal conditions. (**D**) Methylation status of CpG sites in the same region after H37Ra infection for 12 h in both cell types. Methylation percentages are shown below each diagram. Results are representative of three independent experiments. *, *p* < 0.05; **, *p* < 0.01; ***, *p* < 0.001; and ****, *p* < 0.0001.

Bioinformatics analysis also predicted that the G allele of rs1051838 creates a CpG dinucleotide, while the A allele abolishes it. We next investigated whether DUSP14 expression is regulated by DNA methylation. THP-1-derived macrophages and A549 cells were treated with Decitabine, a DNA methyltransferase inhibitor. RT-qPCR results showed that after 72 h of Decitabine treatment, *DUSP14* mRNA levels were significantly increased in both cell types compared with untreated controls (Figure 2A), indicating that DNA methylation suppresses *DUSP14* transcription.

To further test whether methylation in the region surrounding rs1051838 affects transcription, we performed in vitro methylation of the pGL3-rs1051838-G construct using CpG methyltransferase M.SssI, and then transfected the methylated or unmethylated constructs into HEK293T cells. Compared with the unmethylated construct, the methylated construct showed significantly decreased luciferase activity (Figure 2B), confirming that DNA methylation at this locus represses transcriptional activity.

We then examined whether *Mtb* infection affects the methylation status of the rs1051838 region. Bisulfite sequencing PCR (BSP) revealed that under basal conditions, this CpG site was highly methylated in both THP-1-derived macrophages and A549 cells (Figure 2C). Notably, after infection with H37Ra for 12 h, the methylation level in this region decreased in both cell types (Figure 2D), suggesting that *Mtb* infection promotes demethylation at this locus.

Collectively, these data demonstrate that rs1051838 is located within a methylation-sensitive regulatory region, and *Mtb* infection reduces methylation at this locus.

### 3.3. Mtb Infection Upregulates DUSP14 Expression in Macrophages

**Figure 3:**
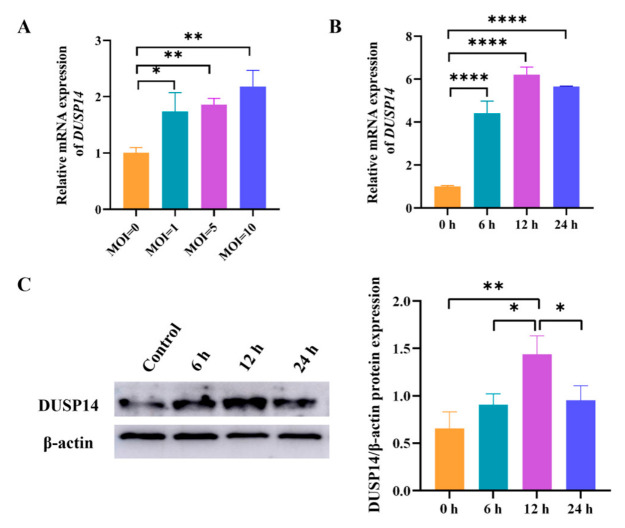
Mtb infection upregulates DUSP14 expression in macrophages. (**A**) *DUSP14* mRNA levels in THP-1-derived macrophages infected with H37Ra at different MOIs (1, 5, or 10) for 6 h. Data are presented as mean ± SD from three independent experiments (*n* = 3). (**B**) Time-course analysis of DUSP14 expression in macrophages infected with H37Ra at an MOI of 10 for 0, 6, 12, or 24 h. DUSP14 mRNA levels were measured by RT-qPCR. Data are presented as mean ± SD from three independent experiments (*n* = 3). (**C**) Time course analysis of DUSP14 protein levels in macrophages infected with H37Ra at an MOI of 10 for the indicated time points. Representative Western blot images are shown on the left, with β-actin as loading control. Densitometric quantification of DUSP14 protein levels from three independent experiments is shown on the right. Data are presented as mean ± SD (*n* = 3). *, *p* < 0.05; **, *p* < 0.01; and ****, *p* < 0.0001.

To determine whether *Mtb* infection affects DUSP14 expression, macrophages were infected with H37Ra at various MOIs (1, 5, and 10), and *DUSP14* mRNA levels were measured by RT-qPCR. The results showed that *Mtb* infection increased *DUSP14* mRNA expression, and the highest expression was observed at an MOI of 10 (Figure 3A). We next performed a time-course experiment at an MOI of 10. Macrophages were infected for 0, 6, 12, or 24 h, and both mRNA and protein levels of DUSP14 were examined. RT-qPCR and Western blot analysis revealed that DUSP14 expression increased after infection and reached its peak at 12 h post-infection (Figure 3B,C). These results indicate that *Mtb* infection upregulates DUSP14 expression in a time-dependent manner.

### 3.4. DUSP14 Regulates Intracellular Mtb Survival and Inflammatory Response in Macrophages

**Figure 4:**
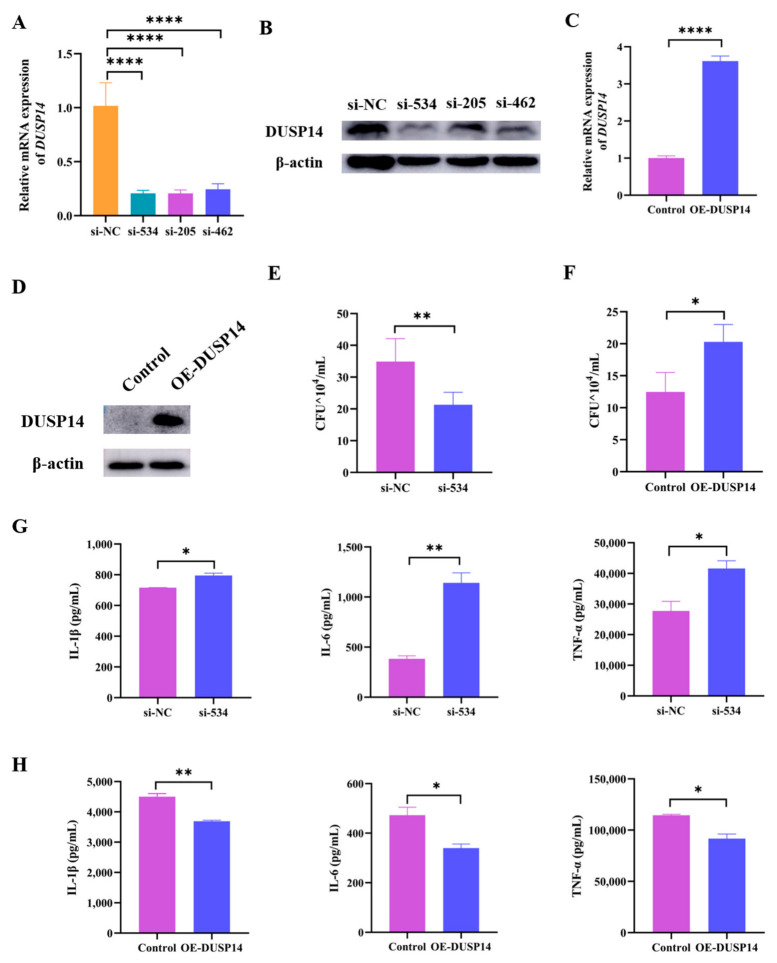
DUSP14 regulates intracellular *Mtb* survival and inflammatory responses in macrophages. (**A**,**B**) Knockdown efficiency of three siRNAs targeting DUSP14 in THP-1-derived macrophages. DUSP14 mRNA levels were measured by RT-qPCR (**A**), and protein levels were detected by Western blot (**B**). si-534 showed the most efficient knockdown and was selected for subsequent experiments. (**C**,**D**) Overexpression efficiency of DUSP14 in THP-1 cells transduced with lentiviral vector. DUSP14 mRNA (**C**) and protein (**D**) levels were significantly increased compared with the control. (**E**,**F**) Intracellular bacterial loads in DUSP14-knockdown (**E**) and DUSP14-overexpressing (**F**) macrophages after H37Ra infection (MOI = 10, 24 h). (**G**,**H**) Cytokine secretion in DUSP14-knockdown (**G**) and DUSP14-overexpressing (**H**) macrophages after H37Ra infection (MOI = 10, 48 h). Levels of IL-1β, IL-6, and TNF-α in culture supernatants were measured by ELISA. Knockdown of DUSP14 significantly increased cytokine secretion, while overexpression significantly reduced cytokine secretion. Data are presented as mean ± SD (*n* = 3). Blots shown are representative of three independent experiments. *, *p* < 0.05; **, *p* < 0.01; and ****, *p* < 0.0001.

To investigate the functional role of DUSP14 in *Mtb* infection, both DUSP14 knockdown and overexpression models were established in THP-1-derived macrophages. For knockdown, three different siRNAs targeting *DUSP14* were tested. RT-qPCR and Western blot showed that si-534 resulted in the most significant knockdown of DUSP14 expression at mRNA and protein levels compared with the control (Figure 4A,B). For overexpression, a lentiviral vector expressing DUSP14 was transduced into THP-1 cells, and the expression of DUSP14 was significantly increased at both mRNA and protein levels compared with the control (Figure 4C,D).

Using above cell models, macrophages were then infected with H37Ra for 24 h and the intracellular bacterial loads were measured. Compared with the control group, knockdown of DUSP14 significantly reduced intracellular bacterial burden (Figure 4E), whereas overexpression of DUSP14 significantly increased intracellular bacterial burden (Figure 4F), indicating that DUSP14 can affect the ability of macrophages to clear intracellular *Mtb*.

To further examine whether DUSP14 regulates pro-inflammatory cytokine production during *Mtb* infection, macrophages were infected by H37Ra for 48 h and IL-1β, IL-6, and TNF-α levels were measured in the culture supernatants by ELISA. In DUSP14-knockdown macrophages, the secretion of IL-1β, IL-6, and TNF-α was significantly higher than that in the control group (Figure 4G). Conversely, in DUSP14-overexpressing macrophages, the secretion of these three cytokines was significantly lower than that in the control group (Figure 4H). Taken together, these data demonstrate that DUSP14 suppresses pro-inflammatory cytokine production and reduces *Mtb* clearance in macrophages.

### 3.5. DUSP14 Negatively Regulates JNK Phosphorylation to Facilitate Immune Evasion During Mtb Infection

**Figure 5:**
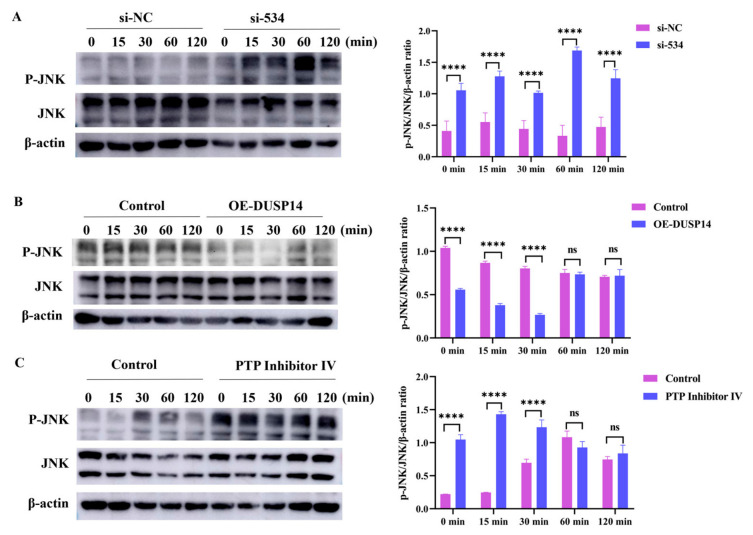
DUSP14 negatively regulates JNK phosphorylation during *Mtb* infection. (**A**) JNK phosphorylation levels in DUSP14-knockdown macrophages at different time points (0, 15, 30, 60, and 120 min) after H37Ra infection (MOI = 10), as determined by Western blot. Knockdown of DUSP14 significantly increased JNK phosphorylation compared with the control. (**B**) JNK phosphorylation levels in DUSP14-overexpressing macrophages after H37Ra infection (MOI = 10) at the indicated time points. Overexpression of DUSP14 significantly decreased JNK phosphorylation compared with the control. (**C**) JNK phosphorylation levels in *Mtb*-infected macrophages treated with or without PTP Inhibitor IV. Treatment with PTP Inhibitor IV significantly increased JNK phosphorylation compared with the untreated control, mimicking the effect of DUSP14 knockdown. Western blots shown are representative of three independent experiments. Data are presented as mean ± SD (*n* = 3). ns, *p* > 0.05; and ****, *p* < 0.0001.

DUSP14 has been reported to dephosphorylate and inactivate JNK [10]. To determine whether DUSP14 regulates JNK signaling during *Mtb* infection, we examined JNK phosphorylation levels in DUSP14-knockdown and DUSP14-overexpressing macrophages at different time points after H37Ra infection (0, 15, 30, 60, and 120 min). Compared with the controls, knockdown of DUSP14 significantly increased JNK phosphorylation (Figure 5A), whereas overexpression of DUSP14 significantly decreased JNK phosphorylation (Figure 5B), indicating that DUSP14 negatively regulates JNK activation during *Mtb* infection.

To determine whether the dephosphorylation of JNK is mediated by the phosphatase activity of DUSP14, we treated *Mtb*-infected macrophages with PTP Inhibitor IV, a small molecule that competitively inhibits DUSP14 enzymatic activity [17]. The treatment with PTP Inhibitor IV significantly increased JNK phosphorylation compared with the untreated control (Figure 5C). This result is reminiscent of the effect of DUSP14 knockdown, indicating that inhibiting DUSP14 activity restores JNK activation. Taken together, these data demonstrate that DUSP14 suppresses JNK phosphorylation in an enzymatic activity-dependent manner, thereby promoting intracellular bacterial survival and reducing pro-inflammatory responses. The DUSP14-JNK axis may represent a potential therapeutic target for host-directed therapy against tuberculosis.

## 4. Discussion

In this study, we found that the non-coding SNP rs1051838 can modulate the functions of macrophages in response to *Mtb* infection by regulating DUSP14 expression, using in vitro models including THP 1-derived macrophages, A549 cells, HEK293T cells, and the attenuated H37Ra strain. rs1051838 functions as a CpG SNP, with the G allele containing a methylatable CpG site that suppresses *DUSP14* transcription upon being methylated. In contrast, the A allele abolishes this CpG site, thus escapes methylation-mediated transcription repression, leading to lower DUSP14 expression and protective effects against TB [8]. Furthermore, we observed that *Mtb* infection can upregulate DUSP14, which negatively regulates JNK phosphorylation in a phosphatase-activity-dependent manner, thereby promoting intracellular *Mtb* survival and dampening pro-inflammatory cytokine production under these experimental conditions. These results provide evidence showing a mechanistic link between genetic variation, epigenetic regulation, and immune function in TB susceptibility in vitro.

The G allele of rs1051838 exhibits stronger transcriptional activity than the A allele in reporter assays. In silico prediction using JASPAR revealed distinct transcription factor binding profiles between the two alleles: the G allele was predicted to bind multiple ETS family transcriptional activators (ETS1, ETV1, ETV4, ETV5, and ETV2), whereas the A allele lost ETS binding but gained repressive SMAD2 and ZBTB family members (ZBTB5, ZBTB26, ZBTB12). ETS proteins promote gene expression by recruiting co-activator complexes [18], and ETS1 binding is methylation-sensitive, requiring a demethylated CpG motif [19,20]. Thus, the G allele supports ETS1-mediated activation, while the A allele recruits SMAD2- and ZBTB-containing repressive complexes that recruit HDACs and establish a silenced chromatin state [21,22]. This “activator versus repressor” model provides a molecular basis for the observed allele-specific transcriptional activity and is consistent with the Vietnamese GWAS reporting that the AA genotype is associated with lower *DUSP14* mRNA levels and protection against active TB [8]. These results suggest an intrinsic allele specific difference in transcriptional activity, with the G allele being more active than the A allele.

Beyond this intrinsic allele-specific difference, we further found that DNA methylation in the rs1051838 region is involved in the regulation of DUSP14 expression. Decitabine treatment increased *DUSP14* mRNA levels, consistent with methylation suppresses *DUSP14* transcription. In vitro methylation of the reporter constructs directly suppressed transcriptional activity, providing evidence that methylation in this region is functionally repressive. Notably, *Mtb* infection reduced methylation across the rs1051838 region and upregulated DUSP14 expression. This observation aligns with reports that *Mtb* actively modulates the host epigenetic landscape through effectors such as Rv2966c and Rv1988 [23,24]. Paradoxically, *Mtb* infection also induces widespread DNA demethylation at host regulatory regions via host demethylases (e.g., TET proteins), which typically precedes transcriptional activation [25,26]. Thus, infection-induced demethylation at the rs1051838 region likely contributes to DUSP14 upregulation.

DUSP14 expression was upregulated upon *Mtb* infection in a time- and MOI-dependent manner. Knockdown of DUSP14 significantly reduced intracellular bacterial burden, whereas overexpression increased bacterial survival, indicating that DUSP14 functions as a host factor hijacked by *Mtb* to promote its intracellular persistence. In parallel, DUSP14 negatively regulated the secretion of pro-inflammatory cytokines IL-1β, IL-6, and TNF-α. These cytokines are critical for bactericidal functions of macrophage, including inducible nitric oxide synthase (iNOS)-mediated nitric oxide production, reactive oxygen species (ROS) generation, and phagolysosomal fusion, as well as for orchestrating protective immune responses such as granuloma formation and Th1 cell differentiation [27,28]. Thus, DUSP14-mediated suppression of these cytokines may contribute to the increased bacterial survival. Interestingly, other DUSP family members have been reported to be implicated in TB-associated immunity. For example, DUSP3 serves as a diagnostic marker for active TB [29], and DUSP12 suppresses pro-inflammatory cytokine production upon BCG infection [30].

PTP Inhibitor IV is a small-molecule inhibitor of DUSP14 enzymatic activity [17]. Consistent with previous reports that DUSP14 directly dephosphorylates and inactivates JNK [31], we found that both knockdown of DUSP14 and PTP Inhibitor IV treatment increased JNK phosphorylation. JNK signaling is known to drive pro-inflammatory cytokine production and antimicrobial responses, including the transcription of TNF-α, IL-1β, and IL-6, as well as the induction of autophagy and apoptosis in infected macrophages [32,33,34]. Taken together, these results suggest a model in which *Mtb*-induced DUSP14 upregulation suppresses JNK activation, thereby reducing cytokine secretion and facilitates intracellular bacterial survival. This DUSP14-JNK axis may represent a critical immune checkpoint exploited by *Mtb* to evade host immunity.

We need to mention several limitations in this study. First, our experiments relied only on THP 1 and A549 cell lines, which may not fully reflect the physiology of primary human macrophages or alveolar epithelial cells. Validation in primary cells or animal models would strengthen the conclusions. Second, PTP Inhibitor IV, used to inhibit DUSP14 activity, may have off target effects on other phosphatases; future studies using DUSP14 specific knockout or phosphatase dead mutant approaches would provide more definitive evidence. Third, although *Mtb* infection reduced methylation across the rs1051838 region, the specific demethylases or methyltransferases responsible for this change remain unidentified. In addition, the transcription factors that preferentially bind the G allele over the A allele and mediate allele specific transcriptional activity have yet to be determined. Fourth, we used the attenuated H37Ra strain in this study; while this strain is widely used for mechanistic studies due to biosafety considerations and ease of handling, it differs from the virulent H37Rv strain in virulence and host immune responses. Therefore, extrapolation of our findings to pathogenic strains should be made with caution. These questions warrant further investigation and are noted as important directions for future studies.

In sum, this study provides mechanistic insights into how rs1051838 regulates DUSP14 expression through allele-specific manner and consequently modulates macrophage anti-mycobacterial immunity in vitro. We demonstrate that the G allele of rs1051838 creates a methylatable CpG site that suppresses *DUSP14* transcription, whereas the A allele escapes this epigenetic repression and is associated with lower DUSP14 expression and protection against TB. Upon *Mtb* infection, pathogen-induced epigenetic remodeling at the rs1051838 region contributes to DUSP14 upregulation, which subsequently suppresses JNK-mediated antimicrobial signaling and pro-inflammatory cytokine production, thereby facilitating intracellular bacterial survival. These findings advance the understanding of host factors contributing to TB susceptibility and suggest DUSP14 as a potential target for host-directed intervention. Small-molecule inhibitors of DUSP14 could potentially enhance JNK-mediated antibacterial functions, which might be beneficial in drug-resistant TB cases where new treatment options are urgently needed [35,36] or as adjunctive agents in combination with existing anti-TB regimens to improve macrophage-mediated bacterial clearance [37]. Future studies in primary cells and animal models will be essential to validate the therapeutic potential of targeting the DUSP14-JNK axis in tuberculosis.

## Data Availability

The original contributions presented in this study are included in the article/Supplementary Materials. Further inquiries can be directed to the corresponding authors.

## Supplementary Materials

The following supporting information can be downloaded at: https://www.mdpi.com/article/10.3390/microorganisms14071588/s1, Table S1. Oligonucleotides and siRNA sequences used in this study. Table S2. JASPAR-predicted transcription factors binding to the rs1051838 G allele. Table S3. JASPAR-predicted transcription factors binding to the rs1051838 A allele.
