# Supplementary material for: rs1051838 Promotes Intracellular Survival of Mycobacterium tuberculosis H37Ra by Regulating DUSP14 Expression
Source: Microorganisms. 2026 Jul 21;14(7):1588. doi: 10.3390/microorganisms14071588 (PMC13413459; doi:10.3390/microorganisms14071588)
Supplement: Supplementary file 1 [file microorganisms-14-01588-s001.zip › Table S1.pdf]

Table S1. Oligonucleotides and siRNA sequences used in this study

Name	Sequence (5’→3’)	Application
DUSP14-rs1051838-F	TCCTTGCCTCCTTGTCTTC	Genotyping
DUSP14-rs1051838-R	TGCTTTATCACCTGTGCCC	Genotyping
M-rs1051838-F	GAGGTCGTAGTGAAAGTCGG	Bisulfite sequencing PCR
M-rs1051838-R	ACATCCGTTCCTCTCACCA	Bisulfite sequencing PCR
GAPDH-F	AGCCTCAAGATCATCAGCAATG	RT-qPCR (internal control)
GAPDH-R	TGTGGTCATGAGTCCTTCCACG	RT-qPCR (internal control)
DUSP14-F	GGACTCTTGAGGAAGAAGGAGAC	RT-qPCR
DUSP14-R	GGAATAGAGAGGAGGTGATTTGAG	RT-qPCR
rs1051838-SacI-F	CGAGCTCTTCCGCTTCCTGGCTCTAT	Cloning into pGL3-basic
rs1051838-HindIII-R	CCCAAGCTTTGCTTTATCACCTGTGCCC	Cloning into pGL3-basic
si-205	Sense: CCAAUUUGAGUAUGUUAAA<br>Antisense: UUUAACAUACUCAAAUUGG	siRNA knockdown
si-462	Sense: GGAGGCAACUGAUAGACUA<br>Antisense: UAGUCUAUCAGUUGUCUCC	siRNA knockdown
si-534	Sense: GCAUAGUUCCCGACGUCUA	siRNA knockdown

Antisense: UAGACGUCGGGAACUAUGC
