# Supplementary material for: rs1051838 Promotes Intracellular Survival of Mycobacterium tuberculosis H37Ra by Regulating DUSP14 Expression
Source: Microorganisms. 2026 Jul 21;14(7):1588. doi: 10.3390/microorganisms14071588 (PMC13413459; doi:10.3390/microorganisms14071588)
Supplement: Supplementary file 1 [file microorganisms-14-01588-s001.zip › Table S2.pdf]

Table S2. JASPAR-predicted transcription factors binding to the rs1051838 G allele

Name	Score	Relative score	Start	End	Strand	Predicted sequence
MA0098.1.ETS1	5.73807	0.905019	8	13	+	GATCCG
MA0145.1.Tfcp2l1	10.0489	0.877812	11	24	+	CCGGACCCAGGCAG
MA0131.3.HINFP	7.33219	0.861388	11	18	-	GGGTCCGG
MA0719.1.RHOXF1	5.28994	0.906247	6	13	-	CGGATCCA
MA0765.1.ETV5	8.15165	0.880864	6	15	-	TCCGGATCCA
MA0764.1.ETV4	7.71807	0.856351	6	15	-	TCCGGATCCA
MA0761.1.ETV1	7.59527	0.855455	6	15	-	TCCGGATCCA
MA1964.2.SMAD2	7.22494	0.902195	11	16	+	CCGGAC
MA1943.2.ETV2::HOXB13	10.991	0.884933	2	14	-	CCGGATCCAGAAA
MA2096.1.ZNF524	9.05854	0.877431	10	18	+	TCCGGACCC
MA1964.1.SMAD2	6.30928	0.857000	9	18	+	ATCCGGACCC
MA2588.1.FAM200B	8.1328	0.866296	11	18	+	CCGGACCC
