# Supplementary material for: rs1051838 Promotes Intracellular Survival of Mycobacterium tuberculosis H37Ra by Regulating DUSP14 Expression
Source: Microorganisms. 2026 Jul 21;14(7):1588. doi: 10.3390/microorganisms14071588 (PMC13413459; doi:10.3390/microorganisms14071588)
Supplement: Supplementary file 1 [file microorganisms-14-01588-s001.zip › Table S3.pdf]

Table S3. JASPAR-predicted transcription factors binding to the rs1051838 A allele

Name	Score	Relative score	Start	End	Strand	Predicted sequence
MA2123.1.Hand1	13.7763	0.968782	10	18	+	TCCAGACCC
MA1964.1.SMAD2	10.6632	0.947047	9	18	+	ATCCAGACCC
MA1964.2.SMAD2	11.5788	1	11	16	+	CCAGAC
MA2509.1.ZBTB5	7.51535	0.856367	6	15	+	TGGATCCAGA
MA1579.2.ZBTB26	9.46886	0.8986	9	16	+	ATCCAGAC
MA1649.2.ZBTB12	7.10585	0.861165	8	14	-	CTGGATC
MA1649.1.ZBTB12	8.26815	0.858575	6	16	-	GTCTGGATCCA
MA1579.1.ZBTB26	8.78742	0.851784	7	21	+	GGATCCAGACCCAGG
MA1153.2.Smad4	8.9712	0.925384	11	17	-	GGTCTGG
MA1153.1.Smad4	9.92739	0.928776	10	17	-	GGTCTGGA
MA0719.1.RHOXF1	5.55961	0.911444	6	13	+	TGGATCCA
MA0145.1.Tfcp2l1	11.1462	0.893791	11	24	+	CCAGACCCAGGCAG
MA0130.1.ZNF354C	5.91578	0.8686938	9	14	+	ATCCAG
MA0092.1.Hand1::Tcf3	9.89686	0.9151715	8	17	-	GGTCTGGATC
MA0092.2.Hand1::Tcf3	9.49642	0.9123686	8	16	-	GTCTGGATC
